# A novel combined systemic inflammation-based score can predict survival of intermediate-to-advanced hepatocellular carcinoma patients undergoing transarterial chemoembolization

**DOI:** 10.1186/s12885-018-4121-3

**Published:** 2018-02-21

**Authors:** Chang Liu, Lei Li, Wu-sheng Lu, Hua Du, Lu-nan Yan, Tian-fu Wen, Wu-ran Wei, Li Jiang, Ming-qing Xu

**Affiliations:** 1 Department of Liver Surgery and Liver Transplantation Center, West China Hospital, Sichuan University, Chengdu, 610041 China; 2 Department of Urology, West China Hospital, Sichuan University, Chengdu, 610041 China

**Keywords:** Hepatocellular carcinoma (HCC), Transarterial chemoembolization (TACE), Derived neutrophil-to-lymphocyte ratio (dNLR), Prognostic nutritional index (PNI), Systemic inflammation

## Abstract

**Background:**

There is currently limited information regarding the prognostic ability of the dNLR-PNI (the combination of the derived neutrophil-to-lymphocyte ratio [dNLR] and prognostic nutritional index [PNI]) for hepatocellular carcinoma (HCC). This study aimed to assess the predictive ability of the dNLR-PNI in patients with intermediate-to-advanced HCC after transarterial chemoembolization (TACE).

**Methods:**

A total of 761 HCC patients were enrolled in the study. The dNLR-PNI was retrospectively calculated in these patients, as follows: patients with both an elevated dNLR and a decreased PNI, as determined using the cutoffs obtained from receiver operating characteristic curve analysis, were allocated a score of 2, while patients showing one or neither of these alterations were allocated a score of 1 or 0, respectively.

**Results:**

During the follow-up period, 562 patients died. Multivariate analysis suggested that elevated total bilirubin, Barcelona Clinic Liver Cancer C stage, repeated TACE, and dNLR-PNI were independently associated with unsatisfactory overall survival. The median survival times of patients with a dNLR-PNI of 0, 1, and 2 were 31.0 (95% confidence interval [CI] 22.5–39.5), 16.0 (95% CI 12.2–19.7) and 6.0 (95% CI 4.8–7.2) months, respectively (*P* < 0.001).

**Conclusions:**

The dNLR-PNI can predict the survival outcomes of intermediate-to-advanced HCC patients undergoing TACE, and should be further evaluated as a prognostic marker for who are to undergo TACE treatment.

## Background

Hepatocellular carcinoma (HCC) is one of the most common malignancies and the third leading cause of cancer-related deaths worldwide [1]. Historically, HCC patients had a very poor prognosis, partly due to generally being at an advanced stage of disease at the time of diagnosis. The curative therapies for HCC remain as liver transplantation, surgical resection, and radiofrequency ablation; however, most patients with HCC are ineligible for these therapies due either to the disease burden or severity of liver disease [2]. For intermediate-to-advanced-stage HCC, transarterial chemoembolization (TACE) is considered the standard treatment by certain international guidelines [3]. The main goal of the above-mentioned treatments is prolongation of life and palliation of symptoms rather than cure; however, further efforts to identify the prognostic factors to better stratify those patients who are likely to benefit from the treatments remain necessary.

Several blood-derived parameters have been demonstrated to be associated with survival of HCC patients. Especially, with the advantage of being readily available from routine tests of blood cell counts and liver function, the derived neutrophil-to-lymphocyte ratio (dNLR) [4, 5] and prognostic nutritional index (PNI) [6, 7] have been widely discussed. The dNLR, a modified score calculated by dividing the neutrophil count by the difference between the white blood cell (WBC) count and the absolute neutrophil count (ANC), has been proposed as an alternative to the NLR in cases in which only the WBC count and ANC have been recorded. A previous study [8] evaluated the prognostic value of the dNLR on outcome in HCC patients following TACE and demonstrated that the dNLR had a similar prognostic value to the well-established NLR; the authors suggested that the dNLR was a cheaper and more easily determinable parameter than the NLR. The PNI, which is calculated from the serum albumin level and total peripheral blood lymphocyte count, was originally proposed to assess the perioperative immunonutritional status and surgical risk in patients undergoing gastrointestinal surgery. Several studies have shown that low PNI relates with poor survival of HCC patients undergoing hepatic resection [6, 9]. However, few data are available for PNI in predicting the clinical outcome of patients with HCC who undergo TACE.

Furthermore, most previous studies focused on either the dNLR or PNI solely, and there are currently few comprehensive studies of the combined use of preoperative inflammation-based prognostic scores and immunonutritional status for survival prediction in HCC patients. Therefore, we hypothesized that a combined systemic inflammation-based grade, namely the dNLR-PNI, which may represent the co-influence of systemic inflammation, may be a more suitable prognostic marker than these two variables alone. Accordingly, in the present study, we aimed to evaluate the prognostic value of the dNLR-PNI grade in patients with intermediate-to-advanced HCC undergoing TACE.

## Methods

### Patients

We retrospectively analyzed a population of HCC patients treated with TACE from 2007 to 2013, at our institution. HCC was diagnosed according to the American Association for the Study of Liver Diseases guidelines [10]. The inclusion criteria were as follows: (1) patients receiving TACE treatment as monotherapy, (2) Child–Pugh liver function of A or B, (3) follow-up time more than 1 month, and (4) unresectable HCC, as determined by multidisciplinary consensus. Major vascular invasion and portal vein thrombosis are not absolute contraindications to TACE at our center. The patients’ demographic and clinical variables and tumor stage were examined.

Written informed consent was obtained from all patients prior to TACE. The study was approved by the independent ethics committees at the West China Hospital, Sichuan University. The study protocol conformed to the ethical standards of the Helsinki Declaration.

### A Novel combined systemic inflammation-based score

The dNLR was constructed as follows: dNLR = neutrophil count to (white cell count-neutrophil count) [4]. The preoperative PNI was calculated using the following formula: serum albumin (g/L) + 0.005 × total lymphocyte count (per mm^3^) [11]. Receiver operating characteristic (ROC) curve analysis was performed to evaluate the sensitivity and specificity of the dNLR and PNI for predicting the 5-year overall survival (OS). The Youden index was estimated to select the optimal cut-off value of dNLR and PNI. A Novel combined systemic inflammation-based score, the dNLR-PNI score, was then calculated by accumulation of the dNLR value and the PNI value as follows: patients with both high dNLR and low PNI were allocated a dNLR-PNI score of 2, while dNLR-PNI scores of 1 and 0 were given to those with either a high dNLR or a low PNI, and to those with both a low dNLR and a high PNI, respectively.

### TACE procedure and follow-up

All TACE procedures were performed by two operators using the same angiographic system (Allura Xper FD20, Philips Healthcare), which has been previously described [12]. Briefly, treatment with TACE was performed with a standard protocol under local anesthesia by 5-fluorouracil (800–1000 mg) and epirubicin-adriamycin (30–40 mg) followed with lipiodol (Lipiodol Ultra-Fluide; Andre Guerbet Laboratories, France) and polyvinyl alcohol foam embolization particles (100–500 μm in diameter; Cook, Bloomington, IN, USA). The chemoembolic agents were injected by percutaneously inserting a microcatheter into the femoral artery of the patient that corresponds to the artery of the liver. When applicable, the artery feeding the tumor was cannulated in a superselective approach. TACE was repeated 4–6 weeks later as needed, until radiographic evidence was obtained of tumor necrosis, tumor progression, or decline in liver function or performance status. OS was defined as the period from the date of first treatment to the date of death, or censorship at the date of last follow-up if the patient is still alive (December 31, 2013).

### Statistics

All continuous variables are listed as the mean ± standard deviation and were compared using one-way analysis of variance. Categorical variables were compared using the χ^2^ test or Fisher’s exact test. OS curves were analyzed using the Kaplan–Meier method and compared using the log-rank test. The Cox proportional hazards model was used for the univariate and multivariate analyses. All factors found to be significant predictors of OS (*P* < 0.10) in the univariate analysis were entered the multivariate analysis. The multivariate analysis was performed using multivariate Cox proportional hazards regression analysis using a forward selection method. The cut-off values for the dNLR and PNI to predict OS were calculated using ROC curves. Additionally, the area under the receiver operating characteristic curve (AUROC) was calculated to compare the discriminatory performance of each scoring system at 1-, 3-, and 5-year intervals. All statistical analyses were performed using IBM SPSS version 24.0 (IBM, North Castle, NY, USA). A two-tailed *P* value of < 0.05 was considered significant.

## Results

### Optimal cut-off values for the inflammation-based index

**Fig. 1:**
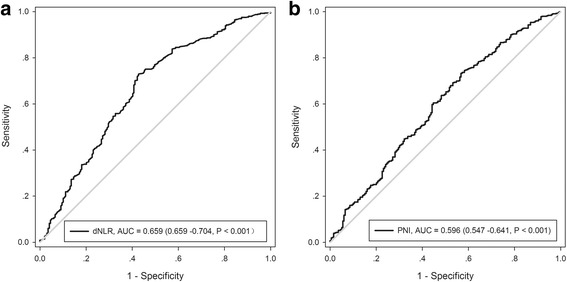
ROC analysis of the preoperative dNLR and PNI value for overall survival. **a** dNLR; **b** PNI

ROC analysis determined optimal cut-off values for dNLR and PNI of 1.7 and 46, respectively, which gave the best sensitivity and specificity for the prognosis of OS, as evaluated by the areas under the curves (Fig. 1). Subsequently, these values were used to calculate the dNLR-PNI score for each patient. Among the 761 HCC patients who received TACE treatment during the clinical course of their disease, 149 (19.6%), 296 (38.9%), and 316 (41.5%) patients were scored as dNLR-PNI 0, 1, and 2, respectively.

### Baseline characteristics

The median values and ranges of the pre-TACE WBC, neutrophil, lymphocyte, and platelet counts, as well as the dNLR and PNI scores, are shown in Table 1. The sample included 643 men (84.5%) and 118 women (15.5%), with a median age of 56 years (range, 19–86 years). Most patients (*n* = 622, 81.7%) were classified as Child–Pugh class A. Hepatitis B infection was the most common etiology of liver cirrhosis (*n* = 650, 85.4%). Overall, 72.0% of the patients received more than one TACE treatment (range: 1–8). No significant differences in age, sex, body mass index, alanine transaminase, tumor number, and model for end-stage liver disease score were observed between the groups. The mean aspartate transaminase level, total bilirubin level, prothrombin time, and largest tumor size were significantly higher in group dNLR-PNI 2 than in the other two groups (*P* < 0.001 for all). The proportions of Child-Pugh grade B, vascular invasion, and patients receiving TACE once were also significantly higher in group dNLR-PNI 2. The relationships between the dNLR-PNI value and the clinicopathological characteristics of the patients are shown in Table 2.

**Table 1:** Values for total white blood cells, neutrophils, lymphocytes, platelet counts, dNLR and PNI

Blood components	Mean	Median	Minimum	maximum	Normal value
Total white blood cells (× 10^9^/L)	5.7 ± 2.7	5.3	3.3	18.2	4.00–10.00
Absolute neutrophil count (×10^9^/L)	4.0 ± 2.4	3.5	1.3	10.7	1.80–6.40
Absolute lymphocyte count (×10^9^/L)	1.2 ± 0.6	1.2	0.4	4.2	1.00–3.30
Total platelets (×10^9^/L)	140.9 ± 89.2	122.5	61	935	100–300
dNLR	2.4 ± 1.5	2.9	0.5	11.5	
PNI	44.9 ± 7.4	44.7	33.1	69.5	

*WBC* white blood cells, *dNLR* derived neutrophil-to-lymphocyte ratio, *PNI* prognosis nutritional index

**Table 2:** Comparison of the clinical characteristics of patients with different dNLR-PNI grade

Variable	dNLR-PNI 0	dNLR-PNI 1	dNLR-PNI 2	*P* value
	(*n* = 149)	(*n* = 296)	(*n* = 316)	
Age (y)	55.8 ± 12.9	54.0 ± 14.0	56.0 ± 13.1	0.15
Gender (male/ female)	131/ 18	252/ 44	259/ 57	0.304
BMI (Kg/m^2^)	22.9 ± 3.1	22.6 ± 3.3	22.3 ± 2.9	0.271
HBsAg (positive/ negative)	133/ 16	242/ 54	275/ 41	0.061
ALT (IU/L)	57.6 ± 49.9	60.9 ± 64.5	57.9 ± 50.8	0.758
AST (IU/L)	58.1 ± 43.1	72.5 ± 64.4	91.3 ± 76.3	< 0.001^b^
TBIL (μmol/L)	18.0 ± 13.3	19.3 ± 11.7	24.7 ± 33.6	0.003^a^
ALB (g/L)	43.1 ± 5.4	40.2 ± 6.1	35.3 ± 4.4	< 0.001^b^
PT (s)	11.9 ± 1.41	12.4 ± 1.9	12.9 ± 1.6	< 0.001^b^
INR	1.1 ± 0.1	1.1 ± 0.1	1.1 ± 0.1	0.175
Creatinine (μmol/L)	78.5 ± 15.6	79.9 ± 65.5	73.5 ± 20.4	0.168
Child-pugh grade (A/ B)	145/ 4	257/ 39	220/ 96	< 0.001^b^
MELD score	5.6 ± 4.3	5.8 ± 5.0	6.3 ± 3.9	0.245
AFP (μg/L), ≤ 400/ > 400	92/ 57	176/ 120	182/ 134	0.69
Largest tumor size (cm)	6.3 ± 3.6	7.4 ± 3.8	9.2 ± 4.7	< 0.001^b^
Tumor number (single/multiple)	61/ 88	119/ 177	133/ 183	0.93
Vascular invasion (absent/ present)	96/ 53	145/ 151	126/ 190	< 0.001^b^
TACE treatments (1/ 2/ > 2)	60/ 38/ 51	128/ 76/ 92	165/ 81/ 70	0.026^a^

*dNLR* derived neutrophil-to-lymphocyte ratio, *PNI* prognostic nutritional index, *WBC* white blood cells, *BMI* body mass index, *ALT* alanine transaminase, *AST* aspartate transaminase, *ALB* albumin, *PT* Prothrombin time, *INR* international normalized ratio, *HBsAg* hepatitis B surface antigen, *AFP* α-fetoprotein, *TBIl* total bilirubin, *MELD* the model for end stage liver disease

^a^*P* < 0.05, when dNLR-PNI 2 vs. dNLR-PNI 0 and dNLR-PNI 1

^b^*P* < 0.05 when each group compared with each other

### Survival analysis

**Fig. 2:**
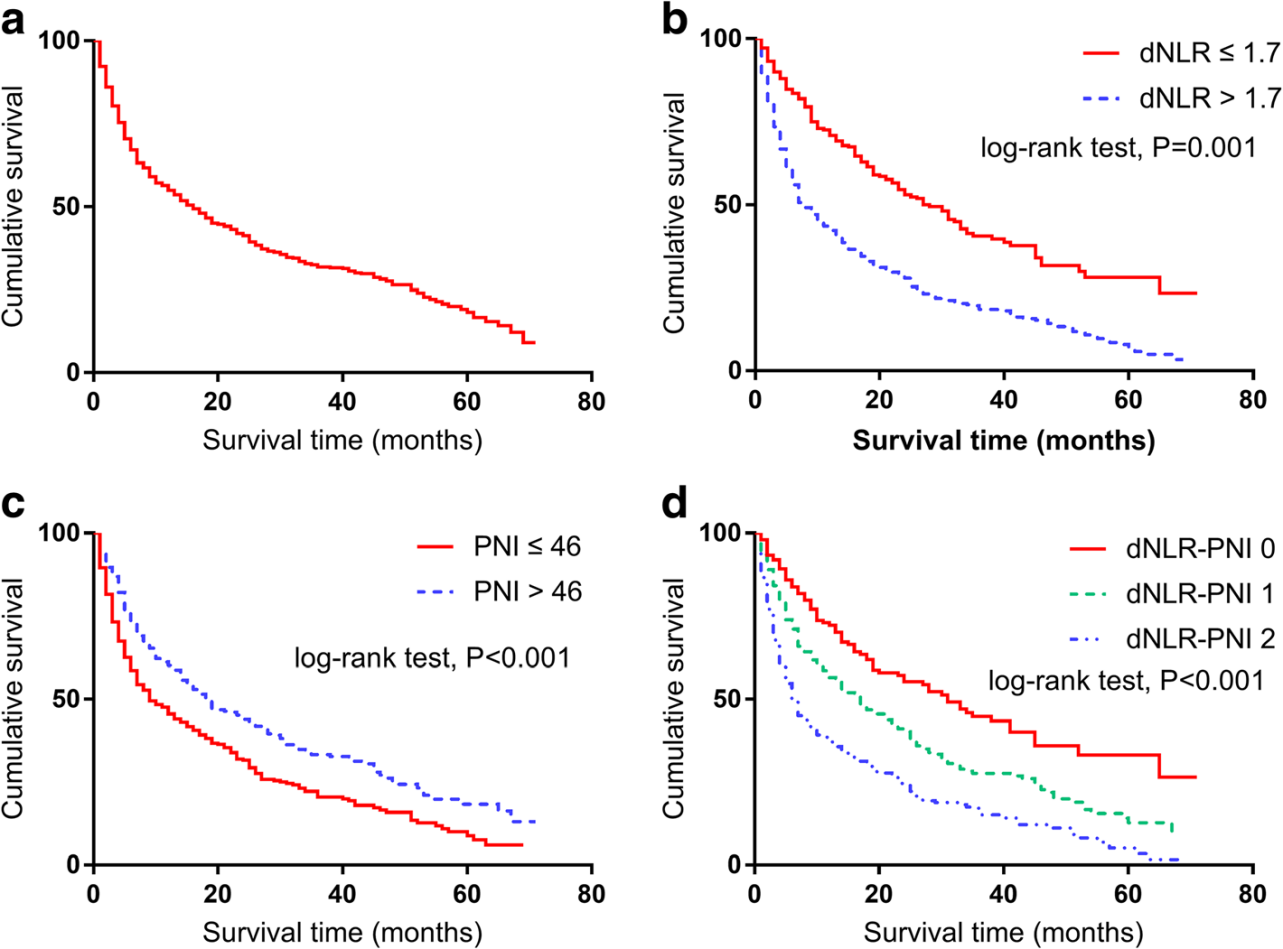
The overall survival rates of the two groups according postoperative prognostic nutrition index. **a** the 1-, 3-, 5- year overall survival rates in group A are 49.7, 25.1 and 12.4%, respectively; **b** the 1-, 3-, 5- year overall survival rates in group dNLR < 1.7 are 69.0, 39.7 and 26.2%, respectively. And, the 1-, 3-, 5-year overall survival rates in group dNLR ≥1.7 are 39.2, 17.1 and 6.0% respectively (*P* < 0.001); **c** the 1-, 3-, 5- year overall survival rates in group PNI < 46 are 42.8, 19.3 and 8.2%, respectively. And, the 1-, 3-, 5-year overall survival rates in group PNI ≥ 46 are 59.0, 32.6 and 17.9% respectively (*P* < 0.001); **d** the 1-, 3-, 5- year overall survival rates in group dNLR-PNI 0 are 71.7, 44.5 and 32.9%, respectively. And, the 1-, 3-, 5-year overall survival rates in group dNLR-PNI 1 are 55.3, 27.0 and 12.4% respectively. And, the 1-, 3-, 5-year overall survival rates in group dNLR-PNI 2 are 34.1, 13.8 and 4.6% respectively (*P* < 0.001)

The median OS for all patients was 12.0 months (95% CI 9.8–14.3); 562 (73.9%) patients died during the follow-up period. The 1-, 3-, and 5-year OS rates were 49.7%, 25.1%, and 12.4%, respectively (Fig. 2a). We compared the OS times in patients with dNLR < 1.7 and dNLR ≥1.7, the optimal cut-off defined using ROC curve analysis, using Kaplan–Meier survival curves. As a result, the median OSs were found to be 27.0 months (95% CI 21.7–32.3) and 7 months (95% CI 5.6–8.4) in patients with dNLR < 1.7 and dNLR ≥ 1.7, respectively (*P* < 0.001; Fig. 2b). With respect to the PNI, the OS times were compared between patients who were categorized as having low and high PNI (cut-off value = 46), demonstrating median survival times of 8.0 (95% CI 5.7–10.3) and 18 months (95% CI 13.8–22.2), respectively (*p* < 0.001; Fig. 2c). In addition, the duration of OS was compared between patients having dNLR-PNI scores of 0, 1, and 2; the median OS times were 31.0 (95% CI 22.5–39.5), 16.0 (95% CI 12.2–19.7), and 6.0 months (95% CI 4.8–7.2), respectively (*P* < 0.001; Fig. 2d).

### Univariate and multivariate analyses

Univariate analysis was performed using a Cox regression model to determine the significant clinicopathological parameters for the prediction of clinical outcomes. The significant factors (*P* < 0.1) in the univariate analysis were further evaluated to determine their influence on OS by multivariate analysis. Our results demonstrated that elevated total bilirubin (hazard ratio [HR] 2.10, 95% CI 1.17–2.29, *P* = 0.004), Barcelona Clinic Liver Cancer stage C (HR 1.78, 95% CI 1.24–2.19, *P* = 0.001), and receiving TACE treatment twice (HR 2.39, 95% CI 1.69–3.38, *P* < 0.001) were independent indictors for worse OS of HCC patients. Moreover, dNLR-PNI 1 (HR 1.71, 95% CI 1.08–2.68, *P* < 0.022) and dNLR-PNI 2 (HR 3.25, 95% CI 1.74–6.02, P < 0.001) were also found to be independent prognostic factors for OS (Table 3).

**Table 3:** Prognostic factors associated with OS

Variables	Univariate		Multivariate	
	HR(95%CI)	*P* value	HR(95%CI)	*P* value
Age (y) (≤ 55, > 55)	0.96(0.81–1.22)	0.413		
Gender (male/female)	1.25(0.89–1.42)	0.319		
HBsAg (positive/ negative)	0.97(0.78–1.22)	0.8		
ALB, g/L (≤ 35, > 35)	0.71(0.59–0.85)	< 0.001		
TBIL, μmol/L (≤ 28, > 28)	1.65(1.33–2.04)	< 0.001	1.64(1.17–2.29)	0.004
ALT, IU/L (≤ 40 > 40)	1.34(1.13–1.59)	0.001		
AST, IU/L (≤ 35 > 35)	2.01(1.64–2.47)	< 0.001		
PT, sec (≤ 12/ > 12)	1.13(0.95–1.34)	0.173		
AFP, ng/mL (≤ 400/ > 400)	1.58(1.34–1.87)	< 0.001		
Child-pugh grade (A/ B)	1.34(1.09–1.66)	0.006		
MELD score (≤ 10/ > 10)	1.45(1.09–1.91)	0.008		
Largest tumor size (cm) (≤ 10/ > 10)	1.75(1.40–2.19)	< 0.001		
Tumor number (single/multiple)	1.01(0.83–1.23)	0.992		
Vascular invasion (present/ absent)	2.02(1.61–2.54)	< 0.001		
BCLC (B / C stage)	2.21(1.76–2.77)	< 0.001	1.65(1.24–2.19)	0.001
dNLR (≤ 1.7, > 1.7)	2.17(1.79–2.61)	< 0.001		
PNI (≤ 46, > 46)	0.66(0.56–0.78)	< 0.001		
TACE treatments (1/ 2/ > 1)				
1	–			
2	1.94(1.58–2.37)	< 0.001	2.39(1.69–3.38)	< 0.001
> 2	1.52(1.21–1.91)	< 0.001	1.39(0.96–2.03)	0.078
dNLR-PNI				
0	–			
1	1.62(1.25–2.09)	< 0.001	1.71(1.08–2.68)	0.022
2	2.75(2.14–3.53)	< 0.001	3.25(1.74–6.02)	< 0.001

*dNLR* derived neutrophil-to-lymphocyte ratio, *PNI* prognostic nutritional index, *WBC* white blood cells, *BMI* body mass index, *ALT* alanine transaminase, *AST* aspartate transaminase, *ALB* albumin, *PT* Prothrombin time, *INR* international normalized ratio, *HBsAg* hepatitis B surface antigen, *AFP* α-fetoprotein, *TBIl* total bilirubin, *MELD* the model for end stage liver disease, *BCLC* barcelona clinic liver cancer

### Discriminatory performances of the staging systems and inflammation scores

**Fig. 3:**
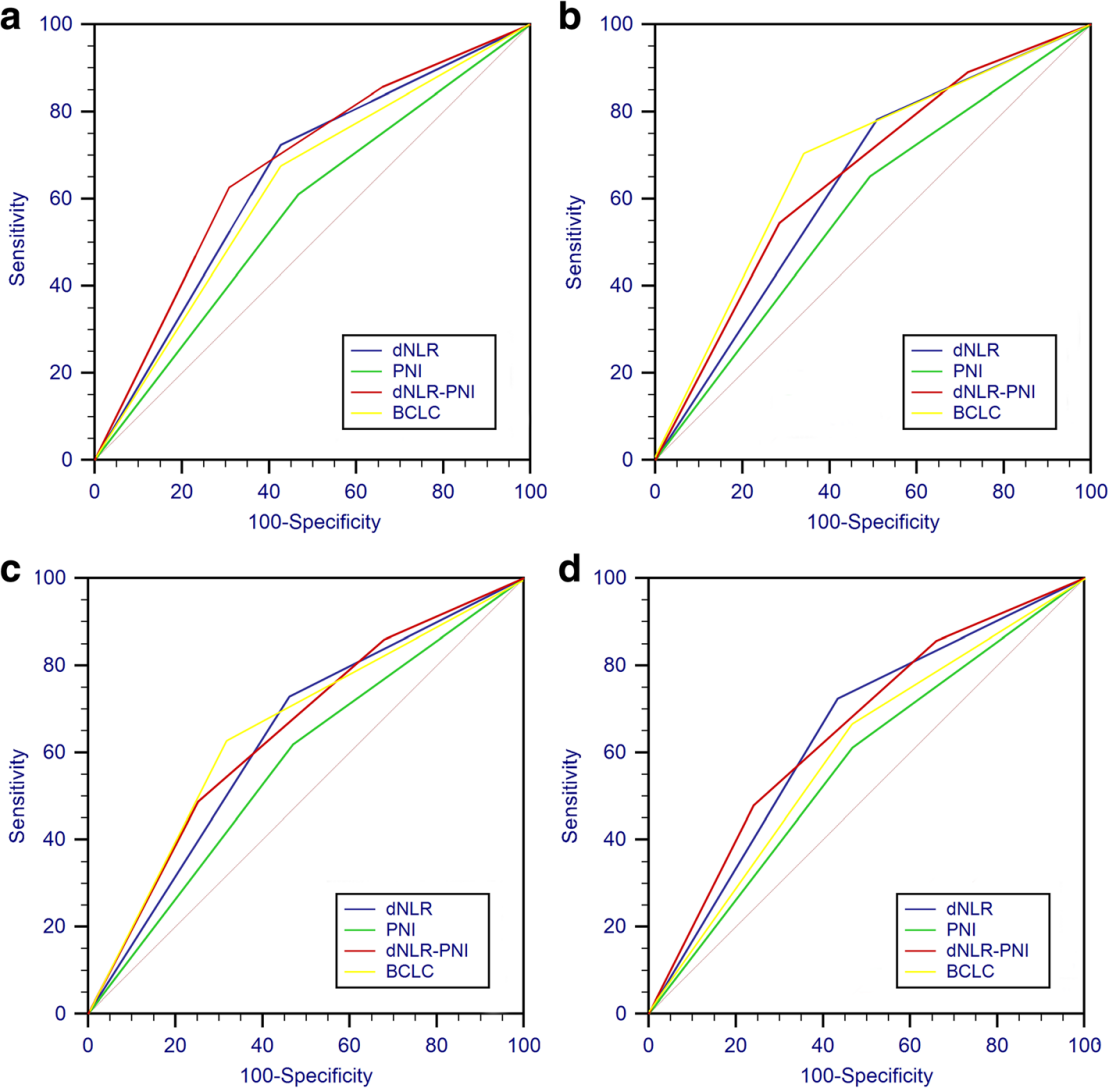
Comparisons of the AUROC values for the survival status between the inflammation-based prognostic scores and the staging systems at overall survive. **a** 1 (**b**), 3 (**c**), and 5-years (**d**)

The discriminatory capacities of each inflammation index, the combined score and BCLC (Barcelona clinic liver cancer) staging system were compared by analyzing the areas under the ROC curves. ROC curves were calculated for the patients’ OS and survival status at the 1-year, 3-year, and 5-year follow-up (Fig. 3). As shown in Table 4, the combined dNLR-PNI score had a better AUROC, however the 95% CI of the AUROC are broadly overlapping with the dNLR and PNI separately.

**Table 4:** Comparison of the AUROC values between the inflammation-based index and grade

period	AUC(95%CI)	*P* value
overall		
dNLR	0.649(0.603–0.694)	< 0.001
PNI	0.571(0.525–0.618)	0.003
dNLR-PNI	0.685(0.633–0.724)	< 0.001
BCLC	0.659(0.611–0.702)	< 0.001
1-year		
dNLR	0.637(0.597–0.676)	< 0.001
PNI	0.579(0.539–0.62)	< 0.001
dNLR-PNI	0.695(0.646–0.724)	< 0.001
BCLC	0.739(0.684–0.761)	< 0.001
3-year		
dNLR	0.634(.590–0.677)	< 0.001
PNI	0.574(0.530–0.618)	0.001
dNLR-PNI	0.679(0.635–0.720)	< 0.001
BCLC	0.652(0.609–0.684)	< 0.001
5-year		
dNLR	0.645(0.600–0.691)	< 0.001
PNI	0.572(0.525–0.618)	0.003
dNLR-PNI	0.683(0.639–0.737)	< 0.001
BCLC	0.594(0.542–0.648)	< 0.001

*dNLR* derived neutrophil-to-lymphocyte ratio, *PNI* prognostic nutritional index, *BCLC* barcelona clinic liver cancer

## Discussion

In this study, we confirmed that the novel combined immunonutritional dNLR-PNI score is an independent predictive factor for prognosis in patients with intermediate-to-advanced HCC undergoing TACE. Patients with dNLR-PNI 2 were associated with a significantly poorer prognosis than their counterparts.

Approximately 70–90% of HCC cases occur in patients with underlying chronic liver disease, including chronic hepatitis B virus infection in eastern Asia and chronic hepatitis C virus infection in European and North American countries, independently from excessive alcohol abuse and metabolic disease [13]. It has become clear that inflammation is central to the pathogenesis of chronic liver injury and has been proposed as a risk factor for HCC. It has been well-established that HCC usually progresses through four stages: cell degeneration, fibrosis, cirrhosis, and tumor formation. Noteworthy, inflammation is involved in all of these stages [14]. With growing evidences regarding the role of inflammation in the HCC pathogenesis, a systemic inflammatory response has been recognized as having prognostic significance in HCC patients after liver transplantation [15], resection [6], ablation, and TACE [16]. The dNLR is composed of only the WBC and neutrophil counts, which is more easily obtained from day-to-day oncological practice, without expensive measurement costs. Proctor et al. [11] first put forward dNLR as an alternative option for clinical trials where only WBC and neutrophil counts were recorded. In their study, 12,118 patients with various kinds of cancers, including colorectal, breast, and lung cancers, among others, were enrolled. They evaluated the prognostic value of the dNLR on OS and cancer-specific survival, and demonstrated that the dNLR had similar prognostic value as the NLR. Furthermore, other researchers have validated elevated dNLR as an independent prognostic factor in patients with pancreatic cancer [17] and colorectal cancer [4]. However, the conclusions of other subsequent studies were inconsistent, with some studies suggesting that the dNLR was not independently associated with survival in patients with renal cell carcinoma [5] and gastric cancer [18]. One possible explanation of these discrepancies among the different studies may be that the use of (WBC - neutrophils) in the denominator of dNLR is broadly mixing two cell types, namely lymphocytes and monocytes, resulting in possible opposing effects in terms of the prognostic value [11]. Lymphocytes have been reported to indicate the generation of an effective anti-tumor cellular immune response [19], while the monocyte count has been demonstrated to be an independent prognostic factor for poor survival in patients with metastatic melanoma [20] and colorectal cancer [21]. Therefore, the inclusion of monocytes may potentially be the reason for these discrepancies.

Malnutrition is a frequently occurring but underdiagnosed problem in both patients with liver cirrhosis and HCC. Patients with HCC are at an especially high risk for malnutrition as the liver is an important metabolic organ, and the majority of cases are associated with liver function impairment due to liver cirrhosis. In addition, tumor progression and tumor therapies can directly impact the liver function [22]. For example, hepatic albumin biosynthesis is downregulated by pro-inflammatory stimuli as part of a negative acute phase reaction in patients with malignancy [23]. Previous studies have demonstrated the independent prognostic value of hypoalbuminemia in HCC [24, 25], and a prospective clinical study revealed malnutrition as an independent negative prognostic risk factor in HCC patients [26]. Accordingly, numerous studies have also shown better recurrence-free survival (RFS) and OS after curative treatment of HCC if the nutritional status was optimized before treatment by supplementation of branched-chain amino acids [27, 28]. In fact, more and more evidence indicated that the cancer cachexia is reflected by a reduction in the level of albumin [29]. Furthermore, some researchers have suggested that a lower pre-treatment PNI could be a visually validated prognostic indicator that predicts an unsatisfied survival [11, 30]. However, other researchers have argued that the preoperative PNI does not affect the postoperative survival outcomes [7]. Further, while an investigation concerning the kinetic changes in the PNI between pre- and post- hepatectomy, rather than the pre-treatment PNI, showed that this index was an independent risk factor for both OS and RFS, this method is not easily available and would therefore not be useful for helping clinicians establishing the appropriate interventions.

As discussed above, with the advantages of being inexpensive and easily available, the dNLR and PNI have been extensively investigated and identified as independent prognostic factors in HCC patients, at least to a certain extent. However, as one factor alone is not sufficient to predict the prognosis accurately, prognostic scores that combine markers of inflammation, such as the dNLR-PNI, are warranted. To the best of our knowledge, this is the first study addressing the prognostic value of a combination of inflammation-based systems in comparison to others prognostic factors in patients treated with TACE treatment. Our results support our hypothesis and indicate that the dNLR-PNI has better discrimination and prognostic abilities compared to the individual indices. Moreover, the results showed that the dNLR-PNI grade was better than presence of vascular invasion and alpha-fetoprotein in predicting poor OS in our cohort. Furthermore, high dNLR-PNI score was related with worse liver function, larger tumor diameter and the presence of vascular invasion, which means that a high dNLR-PNI represents a more aggressive HCC biological phenotype. As mentioned above, low dNLR-PNI score predicts satisfactory survive and multivariate analyses shows its independent prognostic value which validated the dNLR-PNI score as an independent predictor of OS. Our research reflects that the accumulation of two inflammation-based indices is superior to a single inflammation-based index to reflect the systemic inflammatory response for HCC patients receiving TACE treatment.

It is interesting that the numbers of TACE treatments differed according to the dNLR-PNI grade, with having undergone TACE treatment twice found to be a high-risk prognostic factor for OS. In our center, the second TACE was generally performed 1.5–3 months after the initial TACE treatment. Some TACE interventions would be ceased due to tumor progression or a decline in liver function or performance status. In other words, repeat TACE can differentiate patients who did not benefit from the first TACE. HCC patients waiting for liver transplantation usually undergo TACE to downstage the tumor within the Milan criteria and/or as a bridge therapy before liver transplantation; in a previous study, it was identified that patients with a tumor response to TACE treatment had better OS and RFS after liver transplantation [31]. Therefore, HCC progression after TACE is a sign of aggressive tumor behavior, which in turn relates to poor long-term survival status.

There are some limitations in the present study that need to be acknowledged. First, this was a retrospective study, which has inherent limitations and lacking the validation set. Second, it was a single-institution study of a homogenous population. Especially, the patient population is biased due to the high prevalence of hepatitis B virus infection (85.4%). Whether these results can be applied to Western populations wherein hepatitis C virus, nonalcoholic steatohepatitis, and other etiologies of liver disease predominate requires further study and discussion.

## Conclusions

In conclusion, our results revealed that the novel combined inflammation-based immunonutritional score dNLR-PNI is a useful tool in assessing the postoperative survival in intermediate-to-advanced HCC patients after TACE. The dNLR-PNI is simple to calculate from clinical laboratory measures, and is cheap, readily available, and reproducible. Therefore, the dNLR-PNI should be further evaluated as a prognostic marker to predict the outcome of patients with unresectable HCC at the time of diagnosis, as a means to determine the appropriate candidates for TACE treatment.

## Abbreviations

ANC: Absolute neutrophil count
CI: Confidence interval
dNLR: Derived neutrophil-to-lymphocyte ratio
HCC: Hepatocellular carcinoma
HR: Hazard ratio
OS: Overall survival
PNI: Prognostic nutritional index
RFS: Recurrence-free survival
ROC: Receiver operating characteristic
TACE: Transarterial chemoembolization
WBC: White blood cell
